# mTOR-Myc axis drives acinar-to-dendritic cell transition and the CD4^+^ T cell immune response in acute pancreatitis

**DOI:** 10.1038/s41419-020-2517-x

**Published:** 2020-06-02

**Authors:** Dan Xu, Rongli Xie, Zhiwei Xu, Zhifeng Zhao, Min Ding, Wei Chen, Jun Zhang, Enqiang Mao, Erzhen Chen, Ying Chen, Kaige Yang, Tong Zhou, Jian Fei

**Affiliations:** 1 Department of General Surgery, Ruijin Hospital, Shanghai Jiao Tong University School of Medicine, Shanghai, 200025 China; 2 Department of General Surgery, Shanghai 9th People’s Hospital, Shanghai Jiao Tong University School of Medicine, Shanghai, 200011 China; 3 Shanghai 6th People’s Hospital, Shanghai Jiao Tong University School of Medicine, Shanghai, 200233 China; 4 Department of Emergency, Ruijin Hospital, Shanghai Jiao Tong University School of Medicine, Shanghai, 200025 China; 5 Department of Pediatrics, Ruijin Hospital, Shanghai Jiao Tong University School of Medicine, Shanghai, 200025 China

**Keywords:** Stress signalling, Mechanisms of disease, Immunoproliferative disorders

## Abstract

The inflammatory response in acute pancreatitis (AP) is associated with acinar-to-dendritic cell transition. The CD4^+^ T-cell-mediated adaptive immune response is necessary for pancreatic inflammatory damage. However, the effect of acinar-to-dendritic cell transition on the CD4^+^ T-cell response and the regulatory mechanism remain undefined. A mouse animal model of AP was established by repeated intraperitoneal injection of CAE. The mTOR inhibitor rapamycin was administered before AP induction. Primary acinar cells were isolated and co-incubated with subsets of differentiated CD4^+^ T cells. The expression of DC-SIGN was also assessed in pancreatic tissues from human AP patients. We found acinar cells expressed DC-SIGN and displayed the phenotype of dendritic cells (DCs), which promoted the differentiation of naive CD4^+^ T cells into CD4^+^/IFN-γ^+^ Th1 and CD4^+^/IL-17A^+^ Th17 cells in pancreatic tissues during AP. *DC-SIGN* was the target gene of Myc. The mTOR inhibitor rapamycin inhibited AP-induced DC-SIGN expression, CD4^+^ Th1/Th17 cell differentiation and the pro-inflammatory response via Myc. Acinar cells expressed DC-SIGN in pancreatic tissues of human patients with AP. In conclusion, acinar-to-dendritic cell transition is implicated in the CD4^+^ T-cell immune response via mTOR-Myc-DC-SIGN axis, which might be an effective target for the prevention of local pancreatic inflammation in AP.

## Introduction

Acute pancreatitis (AP), triggered by the activation of digestive enzymes in acinar cells, is a clinical cause of acute abdomen^1,2^. AP features local immune dysregulation and pancreatic tissue damage^3,4^. Up to 20% of cases progress to severe AP (SAP), which has a high mortality rate and is associated with pancreatic necrosis, systemic inflammatory response syndrome (SIRS) and multiple organ dysfunction^5,6^. Convincing data have shown that damaged acinar cells induce the initial local inflammatory response^7^, which is accompanied by inflammatory cell infiltration and induction of the T-cell-mediated adaptive immune response^8,9^.

DC-SIGN, also named C-type lectin domain family 4 member L, is the recognition receptor on the surface of dendritic cells (DCs)^10,11^. It has been demonstrated to be implicated in DC migration, T-cell differentiation and pathogen immune escape^12–14^. Accumulating evidence from studies indicates that DC-SIGN is also expressed on gastric epithelial cells, intestinal epithelial cells and tubular epithelial cells, which induces epithelial-DC transdifferentiation and initiates naive CD4^+^ T-cell differentiation^15–17^. Our previous study suggested that AP induces significant upregulation of DC-SIGN in acinar cells, which is associated with the immune response in AP patients, yet the mechanism remains unclear.

Mammalian target of rapamycin (mTOR) is the key molecule that regulates cell growth, proliferation and energy metabolism by sensing the cellular nutrient, oxygen and energy status^18–20^. mTOR functions mainly by forming two complexes: mTOR complex 1 (mTORC1) and mTOR complex 2 (mTORC2), which play different roles in diseases^21^. Previous studies proved that activation of mTOR is involved in the inflammatory response of AP, the progression of chronic pancreatitis and the development of pancreatic ductal adenocarcinomas^22–24^.

Myc is a master transcription factor and is involved in regulating cell proliferation, metabolism and differentiation^25–28^. In pancreatic ductal adenocarcinomas, Myc was found to maintain metabolic homeostasis and cause tumour regression^29^. Myc partially restored the pancreatic parenchyma by inducing re-differentiation of neoplastic ductal cells into acinar-like cells^30^. However, the mechanism by which Myc regulates the phenotype of acinar cells remains undefined.

In this study, we investigated the role of mTOR-Myc signalling in a model of caerulein (CAE)-induced AP. mTOR was activated during AP and this activation induced the level of Myc. Myc transcriptionally regulated DC-SIGN expression on acinar cells and induced acinar-to-DC transition, which promoted type-1 T-helper (Th1) and T-helper 17 (Th17) cell differentiation, inhibited regulatory T cell (Treg) and type-2 T-helper (Th2) cell differentiation, and subsequently induced a local pro-inflammatory response in pancreatic tissues during AP.

## Materials and methods

### Animal models

C57BL/6 mice (8 weeks old) were purchased from Shanghai SLAC Laboratory Animal Co. (Shanghai, China). Mice were housed in a pathogen-free environment and fed standard chow. Animal procedures were approved by the Shanghai Jiao Tong University School of Medicine Institutional Animal Care and Use Committee. The animal sample size (*α* error prob: 0.05; Power: 0,8) was determined using the G*Power software.

GraphPad software was used to randomize mice with a single sequence of random assignments before the treatment. AP was induced using a regimen of 8 hourly intraperitoneal injections of CAE (50 μg/kg; Sigma-Aldrich) for 2 consecutive days^31^. Mice were killed at 12 h, 1 day, 2 days and 7 days after the final CAE injection. Serum and tissues were collected after AP model induction. To inhibit mTOR activity, rapamycin (Rapa; 4 mg/kg/day; Sigma-Aldrich) was administered for 2 days by intraperitoneal injection before the induction of AP. Then, mice were killed at 2 days after the final CAE injection. To inhibit Myc expression, Myc inhibitor 10058-F4 (25 mg/kg) was administered via gavage for 4 days^28^. During the treatments, mice health was monitored constantly. Mice with suffering were discarded from the study. In addition, investigators were blinded to the group allocation during the experiment.

### Human pancreatic specimens

Pancreatic tissue from 100 patients with pancreatitis were obtained from the Emergency Department of Ruijin Hospital. All patient biopsy samples were approved by Ruijin Hospital Ethics Committee. The Ethical Committee decided the sample size. All the patients were enrolled after informed written consent. The pancreatic tissues were collected and immersed in tissue storage solution (Miltenyi Biotec). Then tissues were fixed with 4% paraformaldehyde in phosphate-buffered saline (pH 7.4) and subsequently prepared for immunohistochemical and haematoxylin and eosin (HE) staining.

### Primary acinar cells

Primary acinar cells were isolated from mouse pancreases as previously mentioned^32^. Primary acinar cells were cultured in Dulbecco’s modified Eagle’s medium/F12 and treated with 10^−8^ mol/l CAE for 24 h^33^. We certify that primary acinar cells were screened for *Mycoplasma* contamination. Only *Mycoplasma*-negative primary acinar cells were used in the study.

GraphPad software was used to randomize mice with a single sequence of random assignments before the treatment. Primary acinar cells were transfected with nontargeted control small interfering RNA (siRNA) or si-DC-SIGN using Lipofectamine RNAiMAX (Thermo Fisher Scientific) 24 h before CAE treatment. The sequences of si-DC-SIGN were as follows: mouse DC-SIGN forward, 5′-*GGUUGUCAUCCUUGUCAAAdTdT*-3′ and mouse DC-SIGN reverse, 5′-*UUUGACAAGGAUGACAACCdTdT*-3′. Rapa or 10058-F4 (Selleck) was added 30 min before CAE treatment. Mouse Myc-expressing adenovirus (Ad-Myc) obtained from Hanbio Biotechnology (Shanghai, China) was transfected into primary acinar cells 24 h before CAE administration. The treated acinar cells were collected as conditioned medium (CM) and were co-incubated separately with differently polarized CD4^+^ T cells as described below.

### T-cell differentiation in vitro

Naive CD4^+^ T cells were isolated from spleens using a mouse naive CD4^+^ T-cell isolation kit II (Miltenyi Biotec) and were purified to generate the enriched subsets by flow cytometry^34^. We certify that naive CD4^+^ T cells were screened for *Mycoplasma* contamination. Only *Mycoplasma*-negative naive CD4^+^ T cells were used in the study. Then, naive CD4^+^ T cells were stimulated with anti-CD3 (5 μg/ml, eBioscience) and anti-CD28 (2 μg/ml, eBioscience) antibodies under different polarization conditions to induce differentiation. Naive CD4^+^ T cells were stimulated with interleukin (IL)-12 (10 ng/ml, R&D) in the presence of anti-IL-4 (10 μg/ml, eBioscience) to promote polarization towards Th1 cells. Naive CD4^+^ T cells were stimulated with IL-4 (20 ng/ml, R&D) in the presence of anti-interferon (IFN)-γ (20 μg/ml, eBioscience) to promote polarization towards Th2 cells. Naive CD4^+^ T cells were stimulated with transforming growth factor (TGF)-β (5 ng/ml, R&D) and IL-2 (10 ng/ml, R&D) in the presence of anti-IFN-γ (5 μg/ml, eBioscience) and anti-IL-4 (5 μg/ml, eBioscience) to promote polarization towards Tregs. Naive CD4^+^ T cells were stimulated with TGF-β (2 ng/ml, R&D), IL-6 (30 ng/ml, R&D), IL-1β (10 ng/ml, R&D) and IL-23 (20 ng/ml, R&D) in the presence of anti-IFN-γ (10 μg/ml, eBioscience) and anti-IL-4 (10 μg/ml, eBioscience) to promote polarization towards Th17 cells. Naive CD4^+^ T cells were stimulated with IL-6 and IL-21 (20 ng/ml, R&D), anti-IFN-γ (10 μg/ml, eBioscience) and anti-IL-4 (10 μg/ml, eBioscience) to promote polarization towards follicular helper T (Tfh) cells^35^.

### Flow cytometry

CD4^+^ T cells were isolated from pancreatic tissues and separately stained with antibodies specific for the following molecules: CD4, IFN-γ, IL-4, IL-17A, CD25, Foxp3, CXCR5 and Bcl6 (R&D). Analysis was conducted with FlowJo software (Treestar, Inc., Ashland, OR)^36^.

### Real-time quantitative PCR

Acinar cells were homogenized with Trizol for RNA extraction. Then, RNA was reverse transcribed to cDNA with the GoScript reverse-transcription system (Promega, Madison, WI). Quantitative PCR was performed in an ABI-7900 sequence detection system (Applied Biosystems, Foster City, CA). The results are shown as fold changes with respect to the control^37^.

### Histological examination

HE staining was used to detect tissue injury and was conducted by routine procedures in mouse pancreas tissues and pancreatic biopsy specimens from patients with AP^31^. Anti-DC-SIGN (orb75850, Biorbyt (for mouse pancreatic tissue) and MAI-40070, Thermo Fisher Scientific (for human pancreatic tissue)) antibodies were used. Multiple randomly chosen microscopic fields from at least six mice per group were examined and scored by two pathologists in a blinded manner. The percentage of injured area was calculated on the basis of interstitial oedema and interstitial inflammation, and was used to evaluate the severity of AP^31^.

### Western blotting and ELISA assay

Mouse pancreatic tissues and cell lysates were homogenized in RIPA buffer (Cell Signaling, Danvers, MA) containing a protease inhibitor (Sigma, St Louis, MO). Anti-DC-SIGN (BS70885, Bioworld Technology), anti-mTOR (2972, Cell Signaling Technology), anti-phosphorylated mTOR (P-mTOR; 5536, Cell Signaling Technology) and anti-Myc (ab32072, Abcam) antibodies were used. ImageJ was used to analyze densitometric values on two different scans after background subtraction, from at least three different experiments.

Mouse serum was collected to measure the levels of lipase, amylase and cytokines. Enzyme-linked immunosorbent assays (ELISAs) were performed to measure cytokine levels in animal serum and cell supernatants according to the manufacturer’s instructions.

### Luciferase reporter assay

293T cells were transfected with human Ad-Ctrl or Ad-Myc. Then, luciferase reporter plasmids containing the human wild-type DC-SIGN promoter region (DC-SIGN WT) or a mutated DC-SIGN promoter region (DC-SIGN Mut) were transfected using Lipofectamine^TM^ 2000 reagent. Then, the 293T cells were analyzed via a dual luciferase reporter assay system (Promega, USA)^38^. Luciferase activity of all groups were normalized to *Renilla* luciferase activity and differences between the two groups were indicated as relative fold changes.

### Statistical analysis

Data are presented as the means ± SEMs. Statistical analysis was performed with GraphPad Prism 8 (GraphPad Software, La Jolla, CA). Statistically significant differences were determined by two-tailed Student’s *t*-tests or one-way analysis of variance. All reported data consist of the assumptions of the tests. Test for the assumptions of normality distribution and variance homogeneity have been performed properly. It was used to select the right test for the comparison groups. *P*-values < 0.05 were considered statistically significant.

## Results

### DC-SIGN expression is associated with mTOR activation in AP

**Fig. 1:**
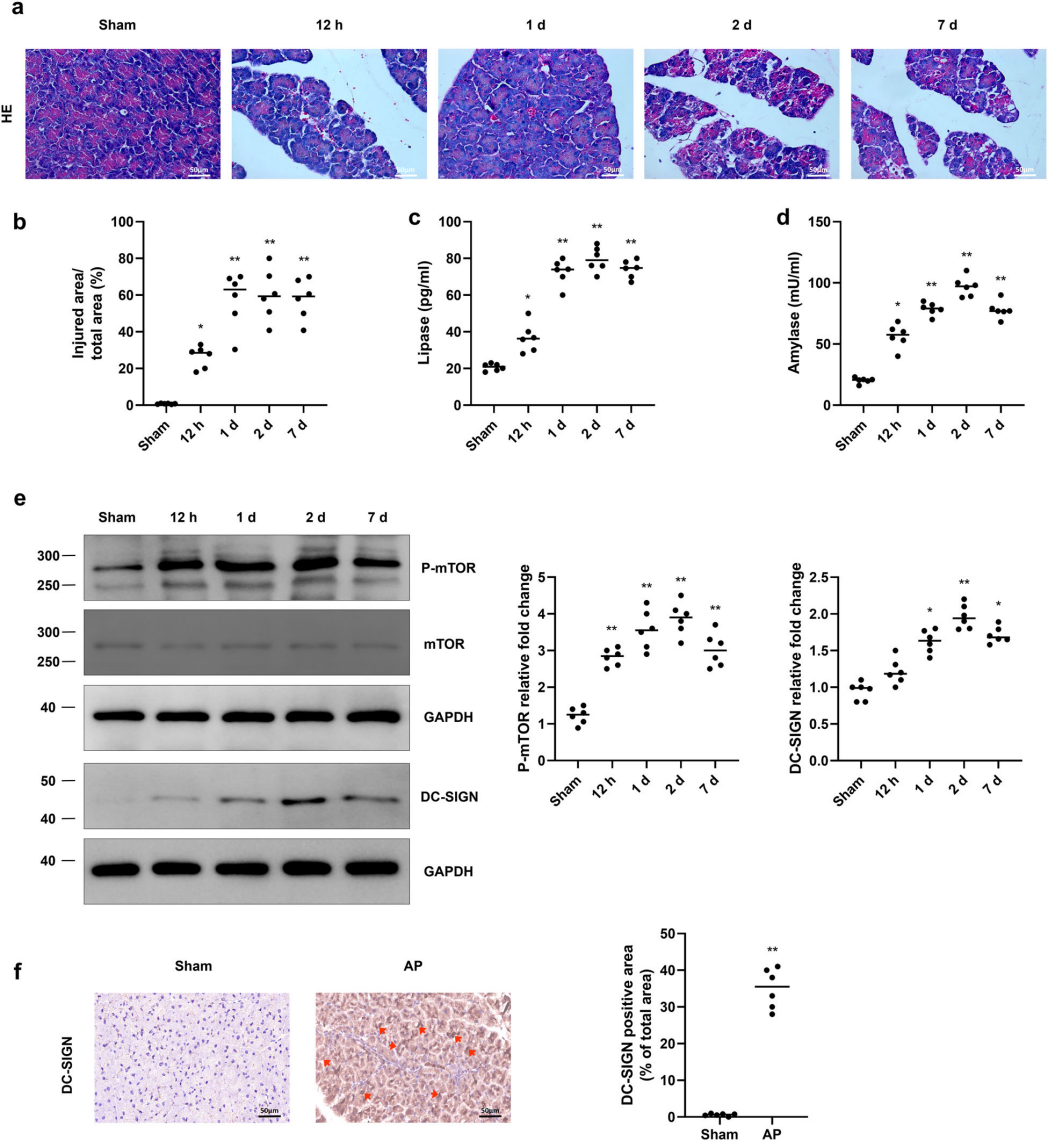
The increase in DC-SIGN expression is accompanied by mTOR activation in AP. **a** Representative images of HE staining of pancreatic tissues from the indicated groups. **b** Quantitative analysis of injured areas in the indicated groups. Serum lipase (**c**) and amylase (**d**) levels were detected by ELISA. **e** Western blot analyses were used to show the levels of DC-SIGN, P-mTOR and total mTOR. **f** Immunohistochemical images were used to visualize the distribution of DC-SIGN in the indicated groups. The red arrows indicate DC-SIGN-positive acini. Quantitative analysis of DC-SIGN-positive areas in the indicated groups. *n* = 6 mice per group. The data are presented as the means ± SEs. *P < 0.05, **P < 0.01 vs. the sham group. AP, acute pancreatitis; P-mTOR, phosphorylated mTOR.

The animal model of AP was established via repeated injections of CAE (50 µg/kg). HE staining of pancreatic tissues revealed that oedema and inflammatory infiltration gradually intensified as AP developed (Fig. 1a, b). As shown in Fig. 1c, d, the serum lipase and amylase activity levels were also increased in AP mice compared with normal mice. The level of P-mTOR was increased 12 h after CAE injection (Fig. 1e). Along with the activation of mTOR, DC-SIGN was subsequently increased on day 1 after CAE injection and peaked on day 2 (Fig. 1e). The histological analysis results further confirmed that acinar cells expressed DC-SIGN at 2 days after the final CAE injection (Fig. 1f). These data show that the DC-like phenotype of acinar cells is connected with mTOR activation and pancreatic injury in the animal model of AP.

### CD4^+^ Th1 and Th17 cell differentiation are induced in pancreatic tissues during AP

**Fig. 2:**
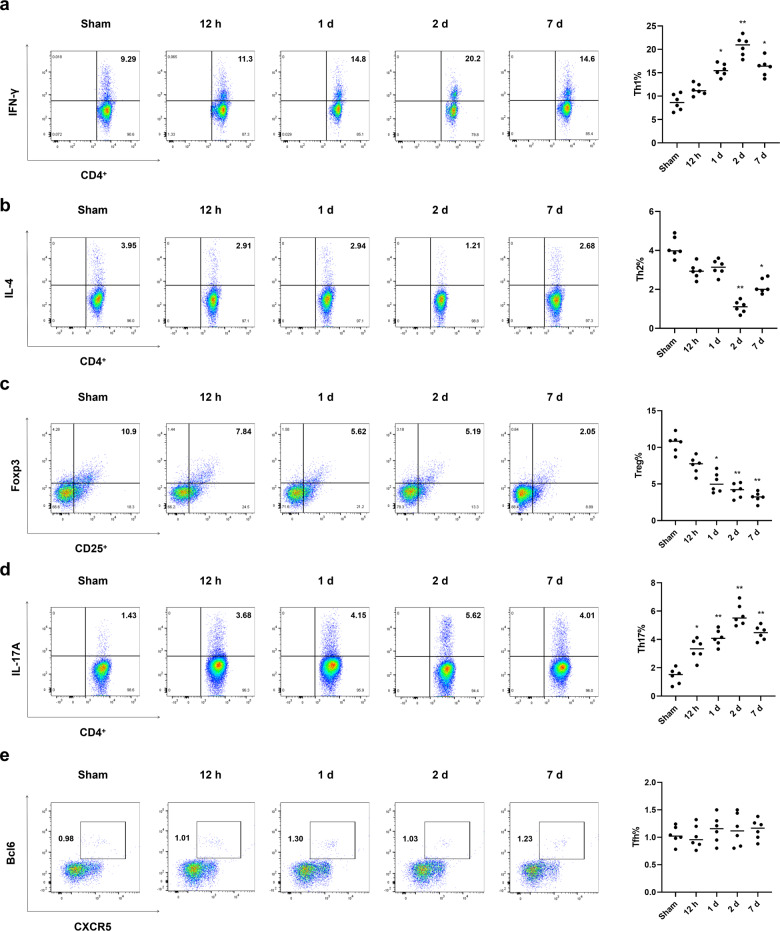
The subset of CD4^+^ T cells is changed in pancreatic tissues after AP. Flow cytometric analysis showed the proportions of CD4^+^/IFN-γ^+^ Th1 cells (**a**), CD4^+^/IL-4^+^ Th2 cells (**b**), Foxp3^+^/CD25^+^ Tregs (**c**), CD4^+^/IL-17A^+^ Th17 cells (**d**), and CXCR5^+^/Bcl6^+^ Tfh cells (**e**). *n* = 6 mice per group. The data are presented as the means ± SEs. *P < 0.05, **P < 0.01 vs. the sham group. AP, acute pancreatitis; Th1, type-1 T-helper; Th2, type-2 T-helper; Tregs, regulatory T cells; Th17, T-helper 17; Tfh, follicular helper T cell.

Subsequently, we used flow cytometric analysis to investigate the time dependence of pancreatic CD4^+^ T-cell subsets. IFN-γ and IL-4 were used to distinguish between Th1 and Th2 (Th1/Th2) differentiation, respectively. We found an increase in CD4^+^/IFN-γ^+^ Th1 cells (Fig. 2a) but a decrease in CD4^+^/IL-4^+^ Th2 cells (Fig. 2b). We quantified the Tregs, which control the balance between the Th1/Th2 responses and immune suppression during the progression of AP. The flow cytometry results exhibited a significant absence of Foxp3^+^/CD25^+^ Tregs after CAE injection (Fig. 2c). In addition, CD4^+^/IL-17A^+^ Th17 cells, which have effects opposite those of Tregs and participate in the maintenance of immune tolerance, were significantly increased at all time points in the animal model of AP (Fig. 2d). We also found that the CXCR5^+^/Bcl6^+^ Tfh cells did not change during AP (Fig. 2e).

Next, an ELISA assay was used to detect cytokines in AP. We found significant increases in IFN-γ (Supplementary Fig. S1a) and IL-17A (Supplementary Fig. S1b), indicating the induction of Th1 and Th17 (Th1/Th17) cell differentiation. The serum cytokine profiles suggested pro-inflammatory characteristics, with elevated levels of IL-6 (Supplementary Fig. S1c) and tumour necrosis factor (TNF)-α (Supplementary Fig. S1d). These results indicate the extensive pro-inflammatory response initiated by Th1/Th17 cells in AP.

### Rapa attenuates the increase in DC-SIGN expression and pancreatic tissue injury in AP

**Fig. 3:**
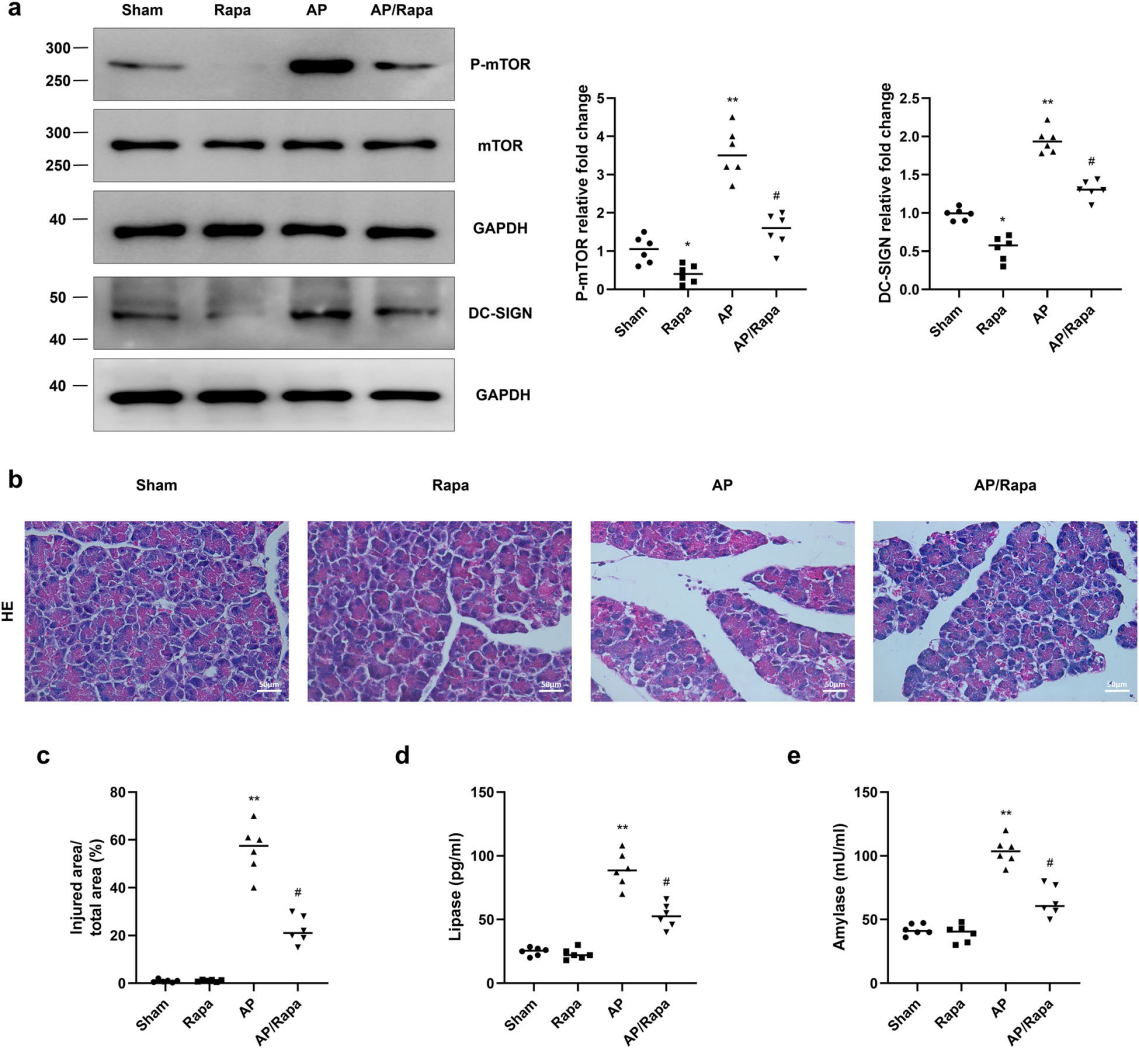
Rapamycin ameliorates AP-induced upregulation of DC-SIGN and pancreatic injury. **a** Protein levels of P-mTOR, total mTOR and DC-SIGN were measured by western blot analysis. **b** HE staining was conducted to assess pancreatic tissue injury in the four groups. **c** Quantitative analysis of the extent of injury. ELISA was used to measure the serum lipase (**d**) and amylase (**e**) levels in the four groups. *n* = 6 mice per group. The data are presented as the means ± SEs. *P < 0.05, **P < 0.01 vs. the sham group. ^#^*P* < 0.05 vs. the AP group. AP, acute pancreatitis; AP + Rapa, rapamycin treatment before the induction of acute pancreatitis; Rapa, rapamycin.

To explore the role of mTOR during AP, Rapa was administered by intraperitoneal injection before AP induction. We found that Rapa inhibited the AP-induced increases in mTOR phosphorylation and DC-SIGN expression (Fig. 3a). In addition, the HE staining results showed that Rapa treatment attenuated aspects of AP-induced pancreatic tissue injury, including pancreatic oedema and inflammatory cell infiltration (Fig. 3b, c). The increases in serum lipase and amylase activity were also inhibited by Rapa treatment (Fig. 3d, e). These results suggest that mTOR-DC-SIGN is necessary for the promotion of pancreatic injury during AP.

### Rapa inhibits AP-induced CD4^+^ Th1/Th17 cell differentiation and the pro-inflammatory response in the pancreas

**Fig. 4:**
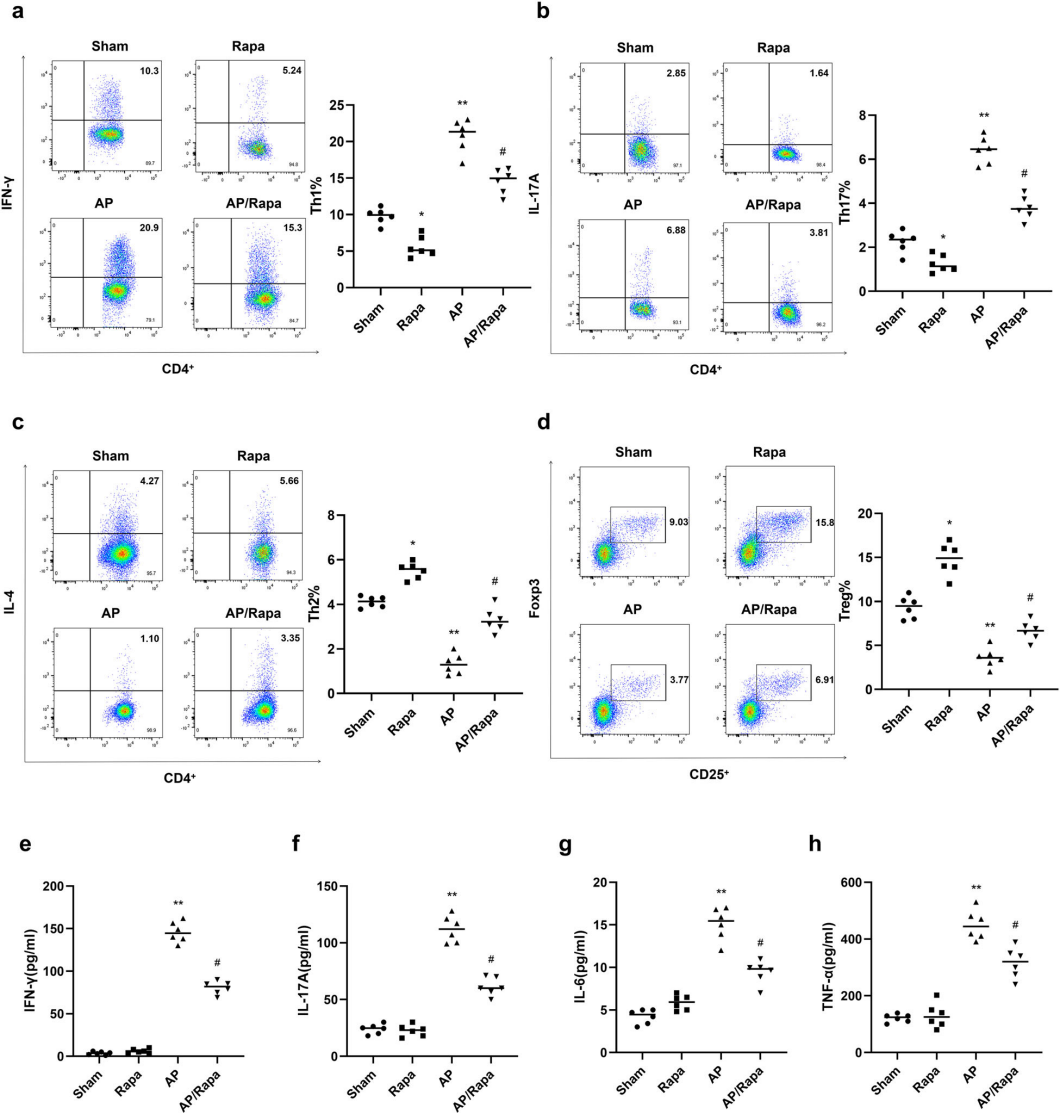
Rapamycin attenuates the AP-induced Th1/Th17-mediated pro-inflammatory response in pancreatic tissues. The proportions of CD4^+^/IFN-γ^+^ Th1 cells (**a)**, CD4^+^/IL-17A^+^ Th17 cells (**b**), CD4^+^/IL-4^+^ Th2 cells (**c**) and Foxp3^+^/CD25^+^ Tregs (**d**) in the four groups were determined by flow cytometry. The serum levels of the cytokines IFN-γ (**e**), IL-17A (**f**), IL-6 (**g**) and TNF-α (**h**) in the four groups were measured by ELISA assay. *n* = 6 mice per group. The data are presented as the means ± SEs. *P < 0.05, **P < 0.01 vs. the sham group. ^#^*P* < 0.05 vs. the AP group. AP, acute pancreatitis; AP/Rapa, rapamycin treatment before the induction of acute pancreatitis; Rapa, rapamycin.

**Fig. 5:**
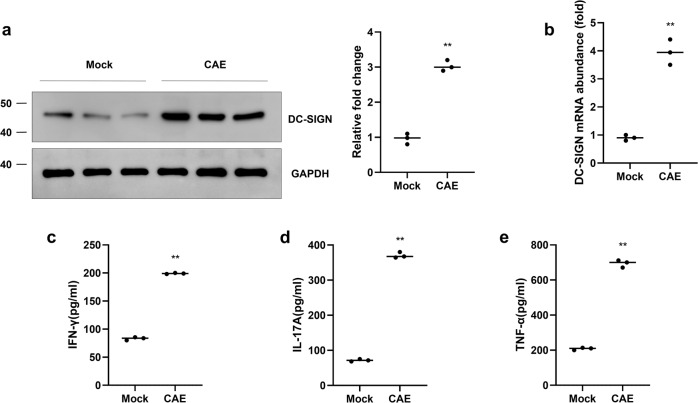
The release of inflammatory cytokines is associated with acinar-to-dendritic cell transition in primary acinar cells. **a** The protein level of DC-SIGN was determined by western blot analysis after caerulein treatment for 24 h. **b** The mRNA level of DC-SIGN was measured in the two groups. An ELISA assay was conducted to measure the IFN-γ (**c**), IL-17A (**d**) and TNF-α (**e**) levels in the supernatant of the two groups. *n* = 3 experimental replicates. The data are presented as the means ± SEs. **P < 0.01 vs. the mock group. CAE, caerulein.

In further experiments, we investigated the mechanism of the AP-induced inflammatory response. The mTOR activity inhibitor Rapa inhibited AP-induced increases in the proportions of CD4^+^/IFN-γ^+^ Th1 cells (Fig. 4a) and CD4^+^/IL-17A^+^ Th17 cells (Fig. 4b). Conversely, Rapa reversed the AP-induced reductions in the proportions of CD4^+^/IL-4^+^ Th2 cells (Fig. 4c) and Foxp3^+^/CD25^+^ Tregs (Fig. 4d). As shown in Fig. 5e–h, the pro-inflammatory cytokines induced by AP—IFN-γ, IL-17A, IL-6 and TNF-α—were strikingly downregulated after Rapa treatment. These results indicate that mTOR signalling regulates the CD4^+^ T-cell-mediated adaptive immune response in pancreatic tissues.

### Acinar-to-DC transition is induced in vitro

To identify acinar-to-DC transition in vitro, primary acinar cells were isolated and the expression of DC-SIGN was detected. The western blotting results showed that CAE treatment induced upregulation of DC-SIGN (Fig. 5a). In addition, the real-time PCR results showed a significant increase in the mRNA level of DC-SIGN after CAE treatment (Fig. 5b). Primary DCs were isolated from mouse bone marrow and induced by granulocyte–macrophage colony-stimulating factor and IL-4. Primary acinar cells were treated by CAE. Traditional DC markers were used to further define the phenotype of DC-like cells. The real-time PCR results showed the upregulation of traditional DC markers in acinar cells after CAE treatment, including MHC-II, CD80, CD86 and CD40. It exhibited a similar transcriptome profiling between CAE-treated acinar cells and primary DCs (Supplementary Fig. 2). The CAE-induced inflammatory response was shown by increases in the supernatant levels of cytokines, including IFN-γ and IL-17, and the inflammatory factor TNF-α (Fig. 5c–e). These results suggest the induction of a DC-like phenotype in acinar cells, which is related to the release of pro-inflammatory cytokines during AP.

### DC-SIGN is implicated in the communication between acinar cells and CD4^+^ T cells

**Fig. 6:**
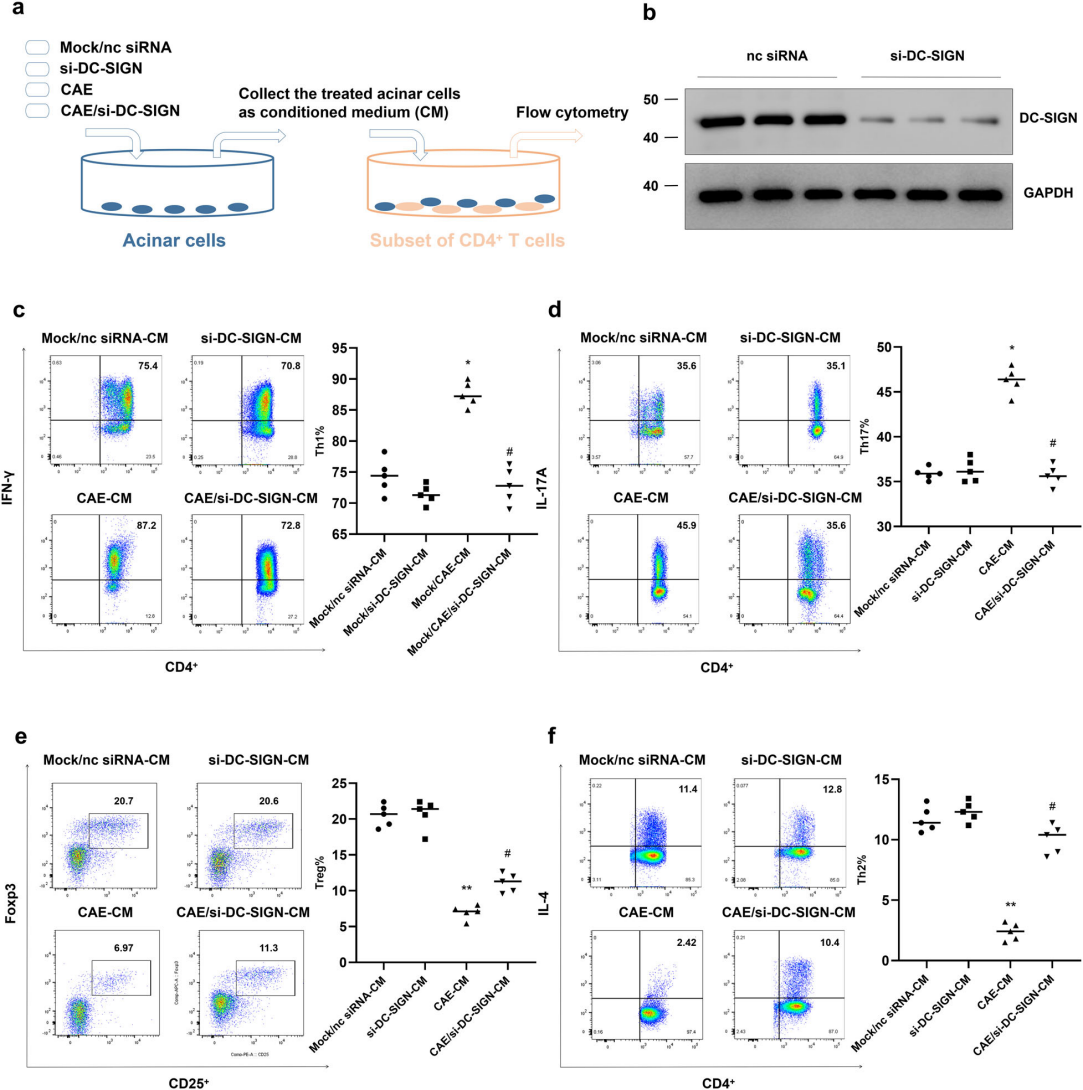
DC-SIGN expressed on acinar cells is responsible for the communication between acinar cells and CD4^+^ T cells. To obtain acinar cells, primary acinar cells were isolated from normal pancreases and transfected with nc siRNA or si-DC-SIGN before caerulein treatment. The treated acinar cells were collected as CM. To obtain subsets of CD4^+^ T cells, naive CD4^+^ T cells were isolated from normal spleens and were induced to differentiate into Th1 cells, Th17 cells, Tregs and Th2 cells in a plate-bound system under different polarization conditions. **a** Schematic showing the co-culture system for acinar cells and subsets of differentiated CD4^+^ T cells. Th1 cells, Th17 cells, Tregs and Th2 cells were co-cultured with Mock/nc siRNA-CM, si-DC-SIGN-CM, CAE-CM and CAE/si-DC-SIGN-CM, respectively. **b** Representative western blotting showing the protein level of DC-SIGN in acinar cells. **c** Flow cytometry was used to determine the proportions of CD4^+^/IFN-γ^+^ Th1 cells (**c**), CD4^+^/IL-17A^+^ Th17 cells (**d**), Foxp3^+^/CD25^+^ Tregs (**e**) and CD4^+^/IL-4^+^ Th2 cells (**f**) under co-culture with Mock/nc siRNA-CM, si-DC-SIGN-CM, CAE-CM or CAE/si-DC-SIGN-CM. *n* = 5 experimental replicates. The data are presented as the means ± SEs. *P < 0.05, **P < 0.01 vs. the Mock/nc siRNA-CM group. ^#^*P* < 0.05 vs. the CAE-CM group. CAE, caerulein; CM, conditioned medium.

To confirm the role of DC-SIGN in the communication between DC-like acinar cells and CD4^+^ T cells, we transfected primary acinar cells with DC-SIGN siRNA before CAE treatment and collected the treated acinar cells as CM (Fig. 6a). In the co-culture system, naive CD4^+^ T cells were isolated from the spleen and were induced to differentiate in a plate-bound system under different polarization conditions. Then, these cells were co-incubated with the different CMs. The western blotting results showed that DC-SIGN was significantly inhibited by si-DC-SIGN transfection in primary cultured acinar cells (Fig. 6b). In CD4^+^ T cells, CAE-CM induced increases in the numbers of CD4^+^/IFN-γ^+^ Th1 cells (Fig. 6c) and CD4^+^/IL-17A^+^ Th17 cells (Fig. 6d), and decreases in Foxp3^+^/CD25^+^ Tregs (Fig. 6e) and CD4^+^/IL-4^+^ Th2 cells (Fig. 6f), but these changes were reversed in the CAE/si-DC-SIGN-CM group. These data provide striking evidence that DC-SIGN expressed on acinar cells is responsible for regulating the differentiation of CD4^+^ T cells in the pancreas.

### mTOR regulates the communication between DC-like acinar cells and CD4^+^ T cells via Myc

**Fig. 7:**
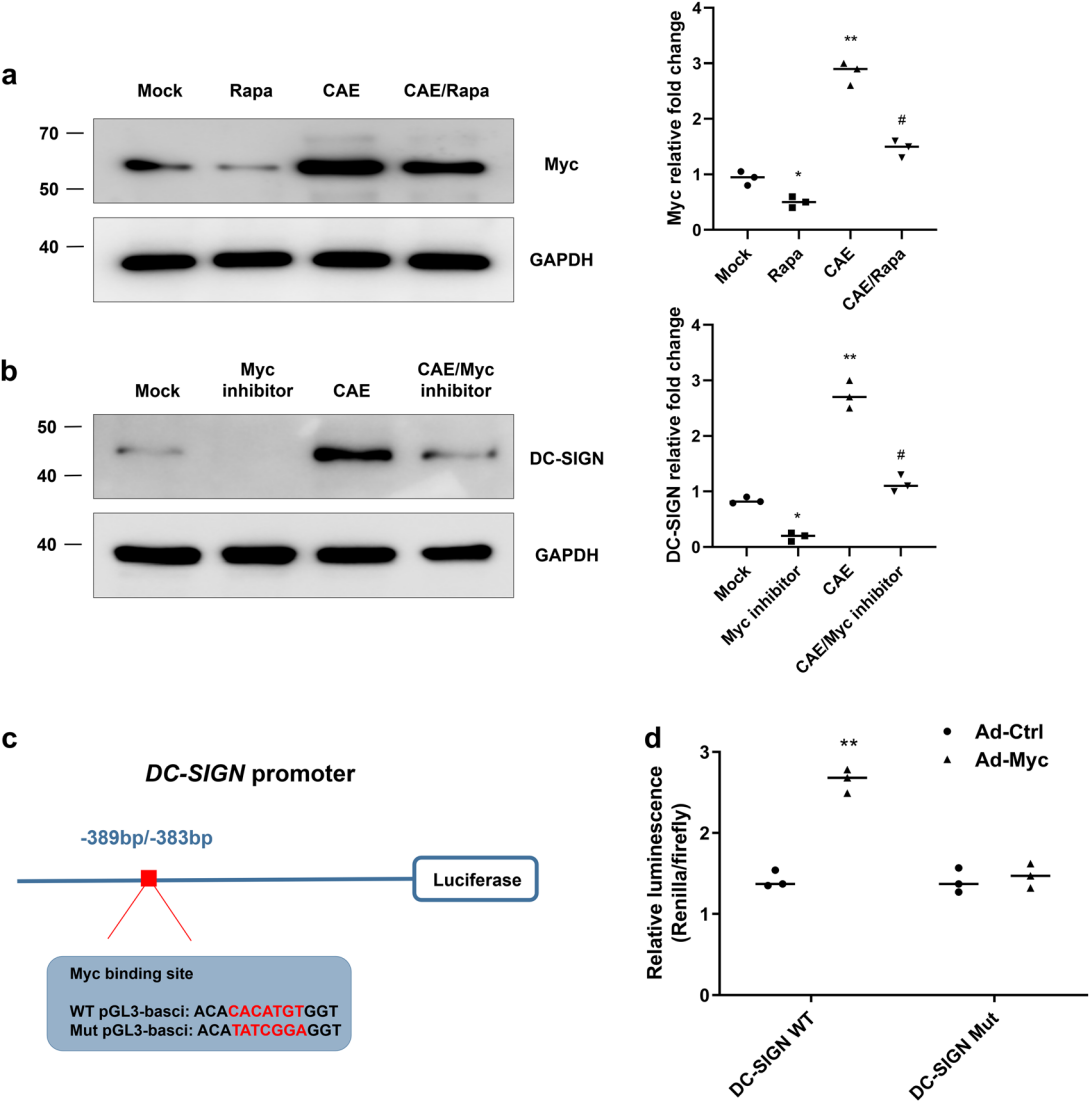
mTOR induces DC-SIGN expression via Myc in vitro. **a** Western blotting showing that rapamycin inhibited the caerulein-induced increase in the Myc protein level. **b** Caerulein-induced DC-SIGN expression was suppressed by treatment with the Myc inhibitor 10058-F4. *P < 0.05, **P < 0.01 vs. the Mock group. #*P* < 0.05 vs. the CAE group. **c** Experimental schematic showing the luciferase vector harbouring the DC-SIGN promoter region. The position relative to the DC-SIGN transcription start site is shown by base pair (bp) numbers. The red box indicates the Myc binding motif. The genomic sequence of Mut pGL3-basic was constructed with a 7 bp mutation (red region) in the WT pGL3-basic site. **d** Luciferase expression was measured in 293T cells from the indicated groups. *n* = 3 experimental replicates. The data are presented as the means ± SEs. *P < 0.05 vs. the Ad-Ctrl group. Rapa, rapamycin; CAE, caerulein.

Next, primary acinar cells were used to investigate the mechanism of DC-SIGN upregulation. The western blotting results showed that Rapa attenuated the expression of Myc induced by CAE (Fig. 7a). Furthermore, the CAE-induced increase in DC-SIGN expression was suppressed by the Myc inhibitor 10058-F4 (Fig. 7b). As shown in Supplementary Fig. S3, these results were confirmed in vivo. To identify whether Myc binds to the DC-SIGN promoter, a luciferase assay was performed. Reconstructed luciferase vectors harbouring either DC-SIGN WT or DC-SIGN Mut (Fig. 7c) were separately transferred into 293T cells after Ad-Ctrl or Ad-Myc transfection. As shown in Fig. 7d, co-transfection of DC-SIGN WT with Ad-Myc increased luciferase activity, but this increase was abolished by co-transfection of DC-SIGN Mut. These data indicate that Myc positively regulates DC-SIGN expression.

**Fig. 8:**
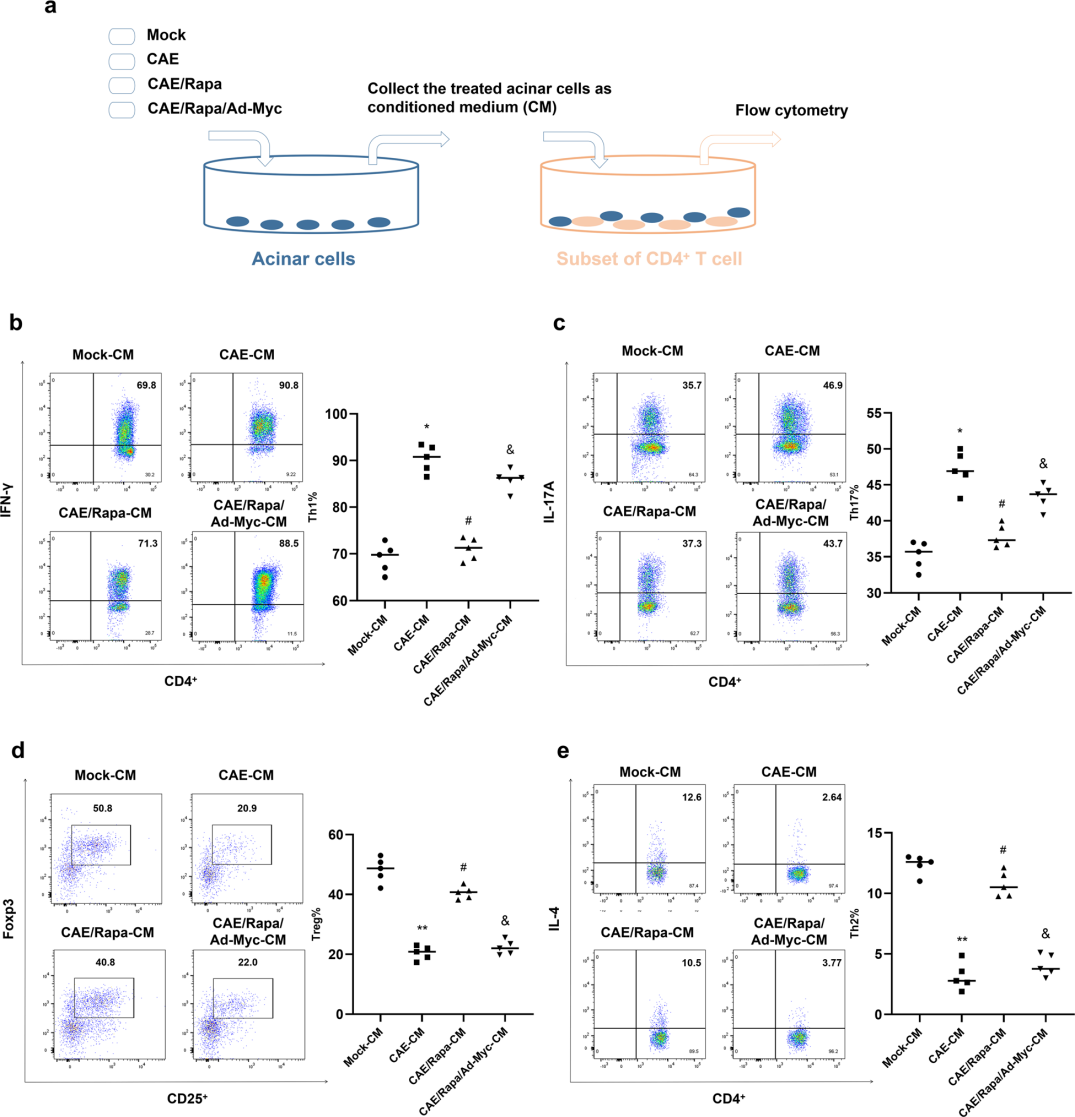
mTOR-Myc signalling is responsible for the communication between DC-like acinar cells and CD4^+^ T cells. **a** Schematic showing the co-culture system for acinar cells and subsets of differentiated CD4^+^ T cells. Th1 cells, Th17 cells, Tregs and Th2 cells were separately co-cultured with Mock-CM, CAE-CM, CAE/Rapa-CM and CAE/Rapa/Ad-Myc-CM. Flow cytometry was used to determine the proportions of CD4^+^/IFN-γ^+^ Th1 cells (**b**), CD4^+^/IL-17A^+^ Th17 cells (**c**), Foxp3^+^/CD25^+^ Tregs (**d**) and CD4^+^/IL-4^+^ Th2 cells (**e**) under co-culture with Mock-CM, CAE-CM, CAE/Rapa-CM or CAE/Rapa/Ad-Myc-CM. *n* = 5 experimental replicates. The data are presented as the means ± SEs. *P < 0.05, **P < 0.01 vs. the Mock-CM group. ^#^*P* < 0.05, ^##^*P* < 0.01 vs. the CAE-CM group. &*P* < 0.05 vs. the CAE/Rapa-CM group. Rapa, rapamycin; CAE, caerulein; CM, conditioned medium.

To determine the role of the mTOR-Myc-DC-SIGN signalling pathway in AP, we transfected primary acinar cells with Ad-Myc before Rapa treatment and collected the treated acinar cells as CM (Fig. 8a). In the co-culture system, CAE/Rapa-CM treatment inhibited CAE-CM-induced increases in CD4^+^/IFN-γ^+^ Th1 cells (Fig. 8b) and CD4^+^/IL-17A^+^ Th17 cells (Fig. 8c), and restored the CAE-CM-induced decreases in Foxp3^+^/CD25^+^ Tregs (Fig. 8d) and CD4^+^/IL-4^+^ Th2 cells (Fig. 8e). In addition, CAE/Rapa/Ad-Myc-CM treatment abolished the effect of CAE/Rapa-CM on CD4^+^ cell differentiation. These data further demonstrate that mTOR-Myc-DC-SIGN signalling in acinar cells is responsible for controlling CD4^+^ Th1/Th17 cell and Treg/Th2 cell differentiation.

### DC-SIGN is expressed on acinar cells in human AP

To evaluate the clinical relationship between DC-SIGN and the pathology of AP, pancreatic biopsy specimens from patients with AP were examined. Acinar cells clearly expressed DC-SIGN from patients with AP (Supplementary Fig. S4a, red arrows). Moreover, HE staining was used to detect the pathological changes in human AP patients. As shown in Supplementary Fig. S4b, the HE staining results suggested a correlation between DC-SIGN expression and pathological pancreatic injury in human AP.

## Discussion

AP is characterized by inflammatory disorder^39^ associated with local pancreatic inflammation that may eventually progress to SIRS^40–42^. Notably, increasing numbers of studies have focused on the role of acinar cells in the initiation of local pancreatic inflammation during AP^43,44^. Our study originally showed that DC-like transdifferentiation of acinar cells initiated CD4^+^ Th1 and Th17 cell immune responses in local pancreatic tissue, and that these responses are regulated by the mTOR-Myc-DC-SIGN signalling pathway in AP.

CD4^+^ T cells are crucial for augmenting the immune response and stimulating pro-inflammatory cytokine production during the progression of AP^45,46^. It has been proposed that the CD4^+^ T cells facilitate the adaptive immune response and determine the disease severity of SAP^47^. Moreover, knockout of CD4^+^ T cells significantly attenuated the pathological changes in SAP. In a recent study, Sendler et al.^48^ reported that the differentiation of CD4^+^ T cells was involved in the development of AP. Increases in Tregs and Th2 cells isolated from the spleens during SAP were identified and these increases induced Treg/Th2 cell-mediated peripheral anti-inflammatory responses within the lymphoid tissue surrounding the pancreas. However, to our knowledge, AP begins with mainly local pancreatic inflammation^44^. In our study, we investigated the local CD4^+^ T-cell immune response by isolating these cells from pancreatic tissues during AP. Th1/Th17 cells were obviously increased and Treg/Th2 cells were concomitantly suppressed in pancreatic tissues. The ELISA results also revealed a Th1/Th17 pro-inflammatory pattern associated with the prominent expression of cytokines, including IFN-γ, IL-17A, IL-6 and TNF-α. These results indicate that the CD4^+^ Th1/Th17 cell-mediated pro-inflammatory response is primarily initiated within local pancreatic tissues during AP and then amplifies the systemic inflammatory response and promotes pancreatic injury.

Communication between DC-like cells and CD4^+^ T cells plays a vital role in promoting inflammation and disease progression^49,50^. In this study, we found that acinar cells expressed DC-SIGN, a signature marker of DC cells, in both the animal model and pancreatic tissues of human AP patients, which participated in the regulation of the CD4^+^ T cell immune response. In a cell co-culture system, knockdown of DC-SIGN on acinar cells attenuated AP-induced CD4^+^ Th1/Th17 cell differentiation and restored CD4^+^ Treg/Th2 cell differentiation. Thus, acinar cells with the phenotype of DCs are the initial source of the regulation of the Th1/Th17-mediated pro-inflammatory response and Treg/Th2 cell-mediated anti-inflammatory response in local pancreatic tissues during AP. It corresponds to the reports that renal tubular cells and podocytes function as DC-like cells^50^.

Myc has been recognized as an important transcription factor associated with the development and differentiation of DCs, DC maturation and metabolism, and the regulatory phenotype of Dex-modulated and MPLA-activated DCs^51–54^. More importantly, Myc expressed by DCs plays an essential role in optimal T-cell priming^55,56^. In this study, we determined the role of Myc in controlling the phenotype of acinar cells in AP. We found that Myc positively regulated the expression of DC-SIGN on acinar cells and induced acinar-DC transdifferentiation, subsequently promoting the differentiation of naïve CD4^+^ T cells into pro-inflammatory Th1 and Th17 cells.

In a recent study, Myc was clarified to activate mTORC1 and to participate in controlling selected amino acid transporters in hepatocellular carcinoma^57^. Rapa is a classical mTOR inhibitor that prevents the assembly of the mTORC1 complex^58^. In our in vivo and in vitro experiments, Rapa suppressed AP-induced DC-SIGN expression by targeting the transcription factor Myc. We characterized the regulatory role of mTORC1 in Myc-driven acinar-DC transdifferentiation in AP. Therefore, our study indicated the regulatory mechanism of DC-SIGN and DC-like functions of pancreatic acinar cells. However, the pattern molecules interacted with DC-SIGN expressed on acinar cells to trigger the DC-like functions is unknown. Further studies are demanded to identify the interaction mechanisms.

In summary, we elucidated that activation of mTOR induced Myc expression, which then transcriptionally upregulated DC-SIGN expression in acinar cells during AP. Activation of mTOR-Myc-DC-SIGN signalling facilitated acinar-to-DC transition, which promoted the pro-inflammatory CD4^+^ Th1/Th17 cell response and suppressed the anti-inflammatory CD4^+^ Treg/Th2 cell response in local pancreatic tissues. Blocking the mTOR-Myc-DC-SIGN axis in acinar cells might be a therapeutic intervention approach by balancing local CD4^+^ T-cell-mediated pro-inflammatory and anti-inflammatory responses during AP.

## Supplementary information

Supplementary Figure Legends

Figure S1

Figure S2

Figure S3

Figure S4
