# Supplementary material for: mTOR-Myc axis drives acinar-to-dendritic cell transition and the CD4+ T cell immune response in acute pancreatitis
Source: Cell Death Dis. 2020 Jun 2;11(6):416. doi: 10.1038/s41419-020-2517-x (PMC7265283; doi:10.1038/s41419-020-2517-x)
Supplement: Supplementary file 1 — Supplementary Figure Legends [file 41419_2020_2517_MOESM1_ESM.doc]

**Supplementary Figure Legends**

**Figure S1.** The serum levels of inflammatory cytokines are increased in AP. An ELISA showed increases in IFN-γ (a), IL-17A (b), IL-6 (c) and TNF-α (d) levels after AP. n = 6 mice per group. The data are presented as the means ± SEs. *P<0.05, **P<0.01 versus the sham group. AP, acute pancreatitis.

**Figure S2.** Caerulein treatment induced the upregulation of traditional DC markers in acinar cells. The mRNA levels of MHC-II, CD80, CD86 and CD40 were measured in the Mock-acinar cells, CAE-acinar cells and primary DCs groups. n = 3 experimental replicates. The data are presented as the means ± SEs. *P<0.05 versus the Mock-acinar cells group. CAE, caerulein; DCs, dendritic cells.

**Figure S3.** mTOR induces DC-SIGN expression via Myc in vivo. Protein levels of Myc and DC-SIGN were measured by Western blot analysis. n = 6 mice per group. The data are presented as the means ± SEs. *P<0.05, **P<0.01 versus the Sham group. #P < 0.05 versus the AP group. Rapa, rapamycin; AP, acute pancreatitis; AP/Rapa, rapamycin treatment before the induction of acute pancreatitis. AP/Myc inhibitor, Myc inhibitor 10058-F4 treatment with acute pancreatitis.

**Figure S4.** DC-SIGN is associated with pancreatitis in human patients with AP. (a) Representative images showing the localization of DC-SIGN in pancreatic specimens from human patients with pancreatitis. The red arrows indicate the with positive DC-SIGN-positive acini. (b) Human pancreatic specimens were subjected to HE staining. AP, acute pancreatitis.
