# Supplementary figures and images for: mTOR-Myc axis drives acinar-to-dendritic cell transition and the CD4+ T cell immune response in acute pancreatitis
Source: Cell Death Dis. 2020 Jun 2;11(6):416. doi: 10.1038/s41419-020-2517-x (PMC7265283; doi:10.1038/s41419-020-2517-x)

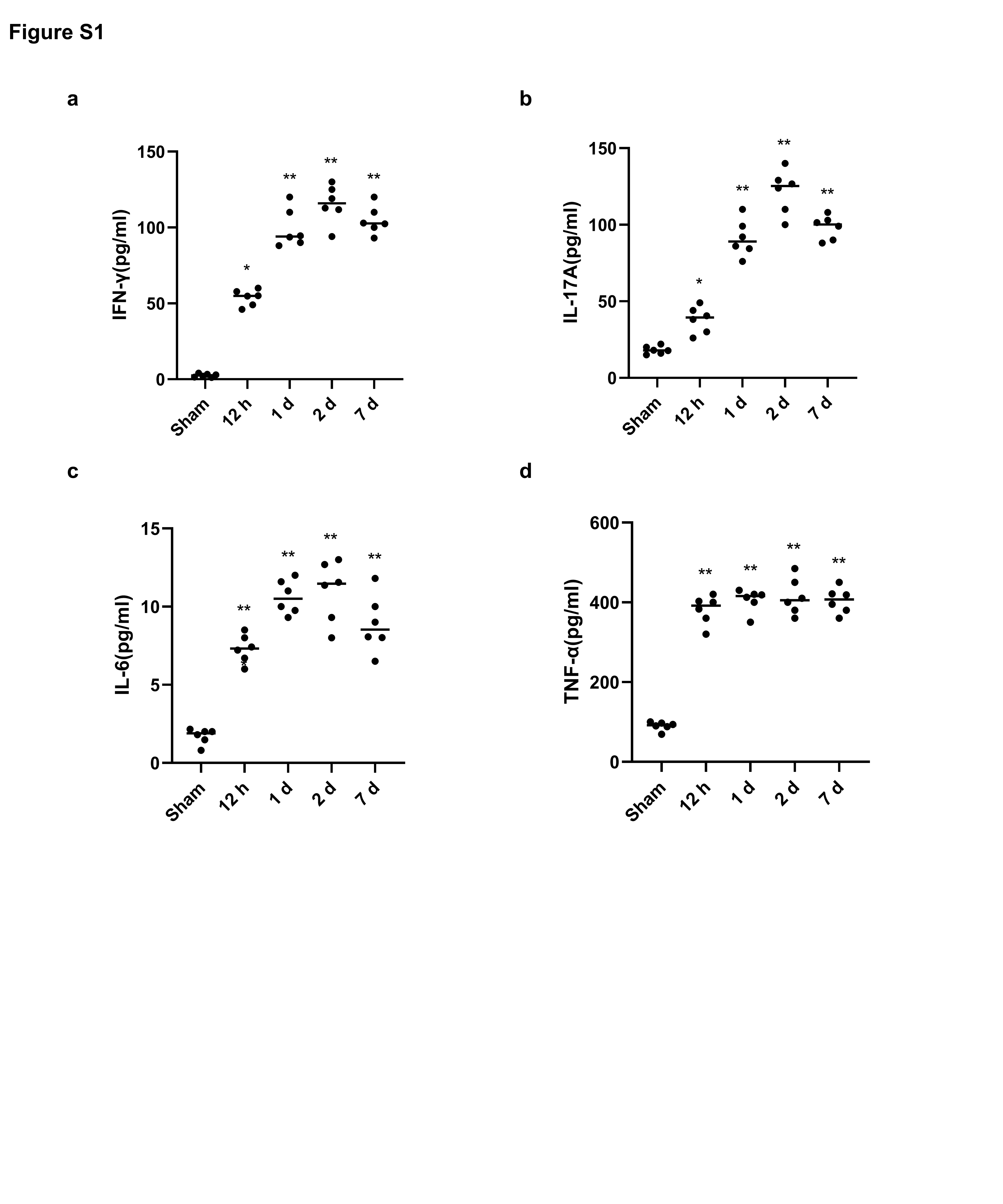

Supplement: Supplementary file 2 — Figure S1 [file 41419_2020_2517_MOESM2_ESM.tif]

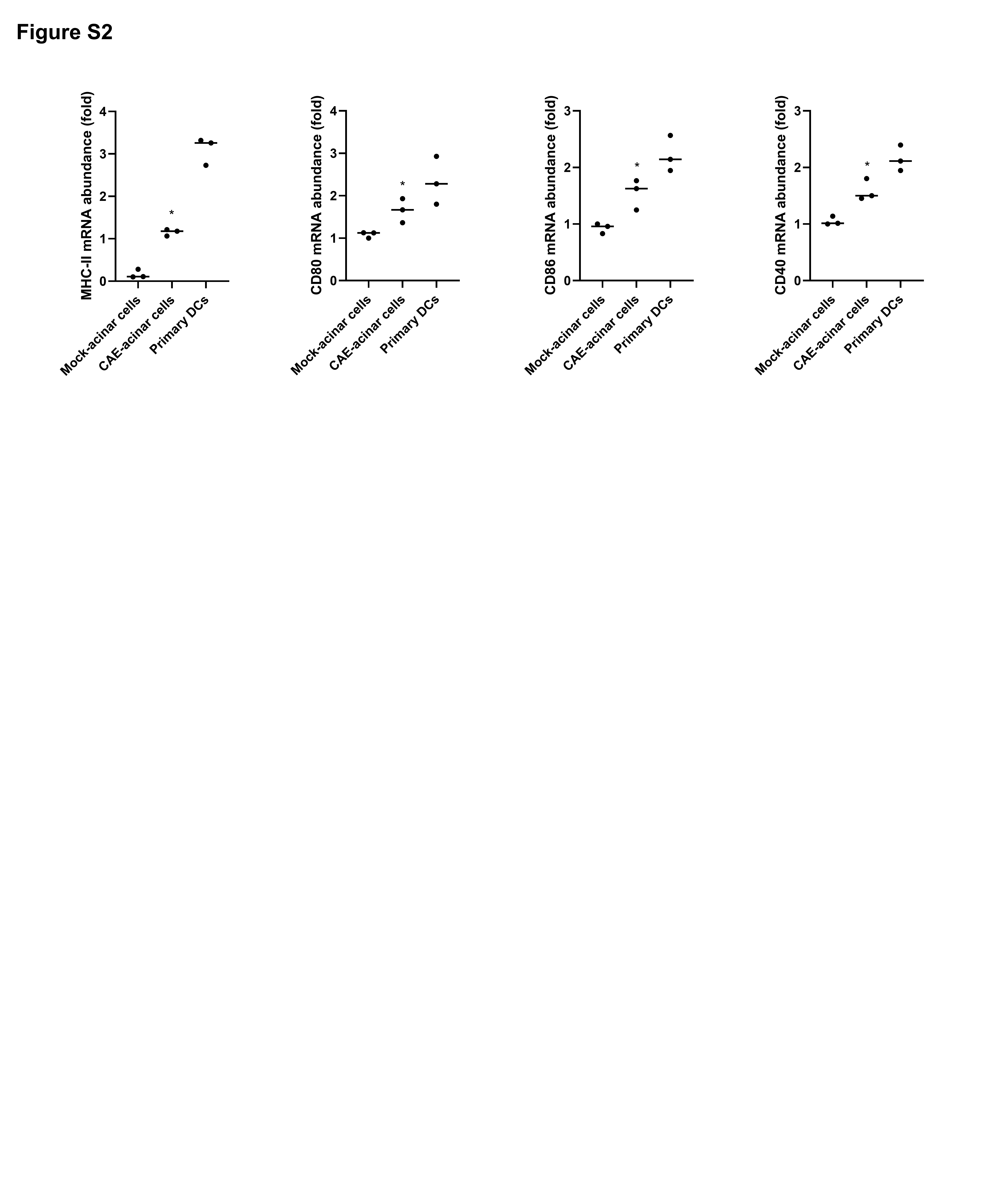

Supplement: Supplementary file 3 — Figure S2 [file 41419_2020_2517_MOESM3_ESM.tif]

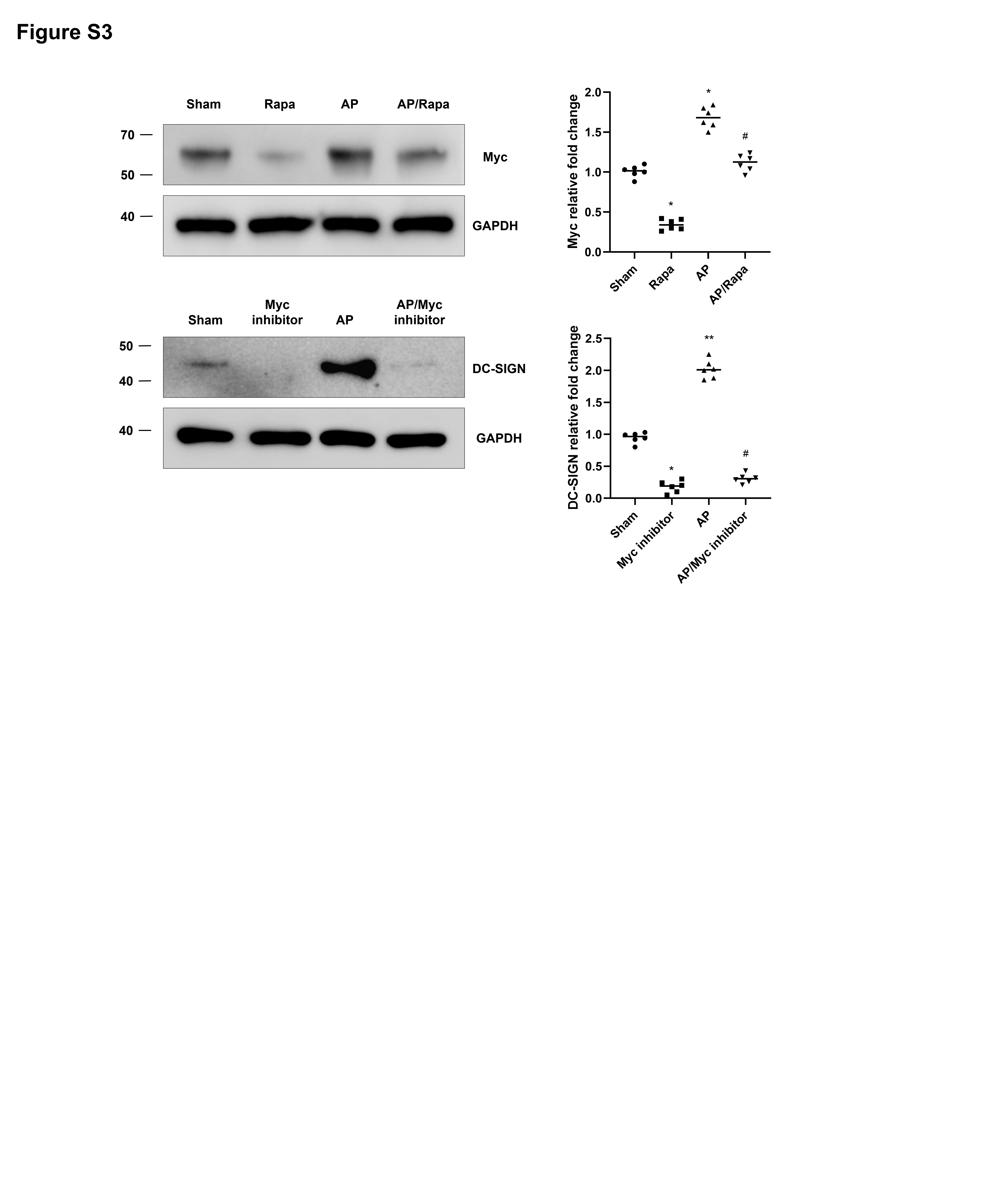

Supplement: Supplementary file 4 — Figure S3 [file 41419_2020_2517_MOESM4_ESM.tif]

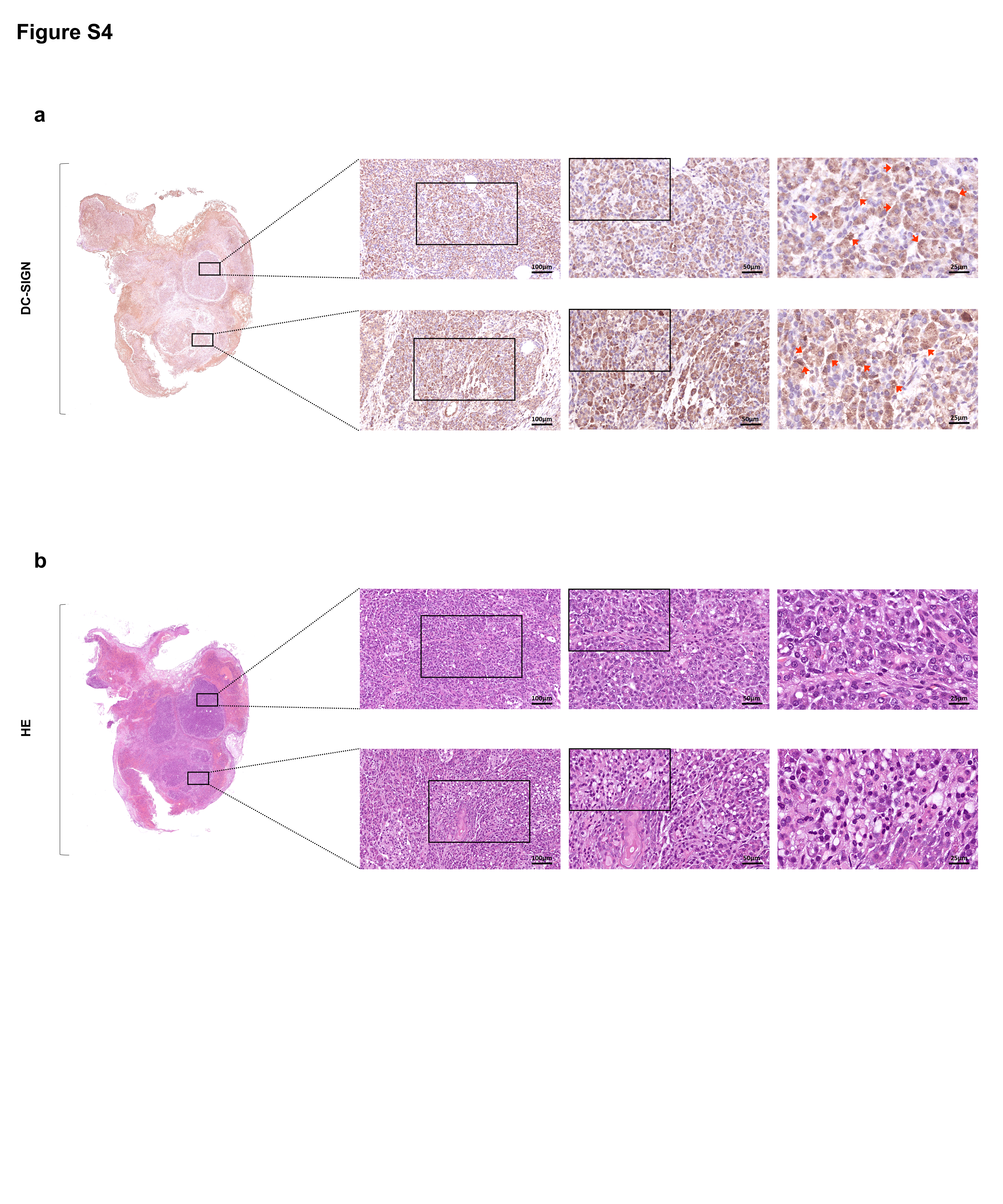

Supplement: Supplementary file 5 — Figure S4 [file 41419_2020_2517_MOESM5_ESM.tif]
